# Investigating the impact of climatic and environmental factors on HFRS prevalence in Anhui Province, China, using satellite and reanalysis data

**DOI:** 10.3389/fpubh.2024.1447501

**Published:** 2024-09-30

**Authors:** Ying Liu, Chengyuan Liu, Liping Wang, Xian Chen, Huijie Qiao, Yan Zhang, Binggang Cai, Rongrong Xue, Chuanxiang Yi

**Affiliations:** ^1^Department of Infection, Yancheng No.1 People's Hospital, Affiliated Hospital of Medical School, Nanjing University, Yancheng, China; ^2^Department of Infectious Diseases, Xuzhou Medical University, Xuzhou, China; ^3^Yancheng Meteorological Administration, Yancheng, China

**Keywords:** HFRS, climatic and environmental factors, satellite and reanalysis data, public health, Poisson regression analysis

## Abstract

**Introduction:**

Hemorrhagic Fever with Renal Syndrome (HFRS) is the most commonly diagnosed zoonosis in Asia. Despite taking various preventive measures, HFRS remains prevalent across multiple regions in China. This study aims to investigate the impact of climatic and environmental factors on the prevalence of HFRS in Anhui Province, China, utilizing satellite and reanalysis data.

**Methods:**

We collect monthly HFRS data from Anhui Province spanning 2005 to 2019 and integrated MODIS satellite datasets and ERA5 reanalysis data, including variables such as precipitation, temperature, humidity, solar radiation, aerosol optical depth (AOD), and Normalized Difference Vegetation Index (NDVI). Continuous wavelet transform, Spearman correlation analysis, and Poisson regression analysis are employed to assess the association between climatic and environmental factors and HFRS cases.

**Results:**

Our findings reveal that HFRS cases predominantly occur during the spring and winter seasons, with the highest peak intensity observed in a 9-year cycle. Notably, the monthly average relative humidity exhibits a Spearman correlation coefficient of 0.404 at a 4-month lag, taking precedence over other contributing factors. Poisson regression analysis elucidates that NDVI at a 2-month lag, mean temperature (T) and solar radiation (SR) at a 4-month lag, precipitation (P), relative humidity (RH), and AOD at a 5-month lag exhibit the most robust explanatory power for HFRS occurrence. Moreover, the developed predictive model exhibiting commendable accuracy.

**Discussion:**

This study provides key evidence for understanding how climatic and environmental factors influence the transmission of HFRS at the provincial scale. Insights from this research are critical for formulating effective preventive strategies and serving as a resource for HFRS prevention and control efforts.

## 1 Introduction

Hemorrhagic Fever with Renal Syndrome (HFRS) is the most commonly diagnosed zoonosis in Asia. This zoonotic infection is caused by exposure to aerosols contaminated with the virus. For instance, infection with Ortho hantavirus can induce HFRS, a condition characterized by acute kidney injury and increased vascular permeability (1). Ortho hantavirus is typically transmitted to humans through the inhalation of, or contact with, rodent excreta such as urine, feces, and saliva. The clinical manifestations of this infection include fever, hemorrhage, headaches, back pain, abdominal pain, severe renal failure, and hypotension (2). The disease progresses through five distinct stages: the febrile phase, hypotensive shock phase, oliguria phase, polyuria phase, and recovery phase. Currently, HFRS poses a serious threat to public health and economic development in more than 30 countries worldwide (3). Despite preventive measures such as vaccination, environmental management, and systematic rodent control, HFRS remains prevalent across multiple provinces, cities, and autonomous regions in China (4). The high incidence rate in China underscores the considerable risk it poses to public health (5).

Rodents, as the primary hosts of HFRS, play a crucial role in the natural occurrence of this epidemic disease. The prevalence of HFRS is shaped by a combination of natural factors, including climate, environment, and landscape, as well as social factors such as population dynamics, economic development, healthcare conditions, immunization practices, and their interactions (4, 6). Among these factors, climatic and environmental elements such as temperature, humidity, precipitation, sunlight, air pollution, and the normalized difference vegetation index (NDVI) significantly influence disease transmission. These elements create ecological conditions that allow pathogens, animal hosts, and vectors to thrive, thereby affecting the epidemiological characteristics of HFRS (5–7).

Despite the recognized influence of these factors, there is still no consensus on their exact impact on the disease, as some studies have reported contradictory findings (6, 8). For instance, the relationship between precipitation and disease incidence has been reported as positive (2, 5, 9), negative (7, 10, 11), or not significantly correlated (12). Rats, which thrive in marshes and low-lying areas with sufficient moisture, May influence disease transmission in the following months due to favorable conditions for their reproduction. Precipitation is linked to overall health indicators and greenery, which in turn affect rat habitat and food availability, thereby influencing the likelihood of disease transmission. Similarly, studies on temperature-related indicators, including mean, minimum, and maximum air temperatures as well as surface temperatures, have yielded inconsistent results (2, 5, 12, 13). Although these findings vary, the prevailing view is that rats and hantaviruses thrive most actively within a temperature range of 10 to 25°C (6). Research on humidity, on the other hand, has been more consistent, with most studies identifying it as a protective variable against HFRS occurrence (2, 12–15). Moreover, recent studies on external factors influencing HFRS prevalence (11, 16) have revealed that variables such as temperature, relative humidity, precipitation, solar radiation hours, air quality, and NDVI are all associated with the frequency of HFRS cases. Additionally, recognizing the latency period in the infection’s replication and spread, which often results in a lag effect of 1 to 6 months for climatic and environmental conditions, is crucial for accurate analysis (2).

However, it should be noted that the majority of aforementioned studies rely on meteorological station measurements, which provide only small-scale observations. This approach poses challenges in accurately capturing broader spatial and temporal patterns, leading to limitations in research findings and restricting the applicability of these conclusions to larger scales, such as city or provincial levels. Alternatively, the use of remote sensing technology, which can gather extensive data with varying geographical and temporal characteristics, offers a promising solution. The objective of this study is to gain a comprehensive understanding of how climatic and environmental factors influence the epidemiological characteristics of HFRS at a larger scale. To achieve this, we utilize remote sensing and reanalysis data to establish quantitative statistical relationships between climatic factors (e.g., precipitation, temperature, humidity, and solar radiation) and environmental factors [e.g., aerosol optical depth (AOD) and NDVI] with the epidemiological characteristics of HFRS in Anhui Province, eastern China. The findings are expected to provide valuable theoretical insights for medical professionals, enhancing the understanding of HFRS epidemiological characteristics and supporting the development of predictive models to forecast HFRS frequency. Such insights will be critical for formulating effective preventive strategies and serving as a resource for HFRS prevention and control efforts.

## 2 Materials and methods

### 2.1 Study area

**Figure 1:**
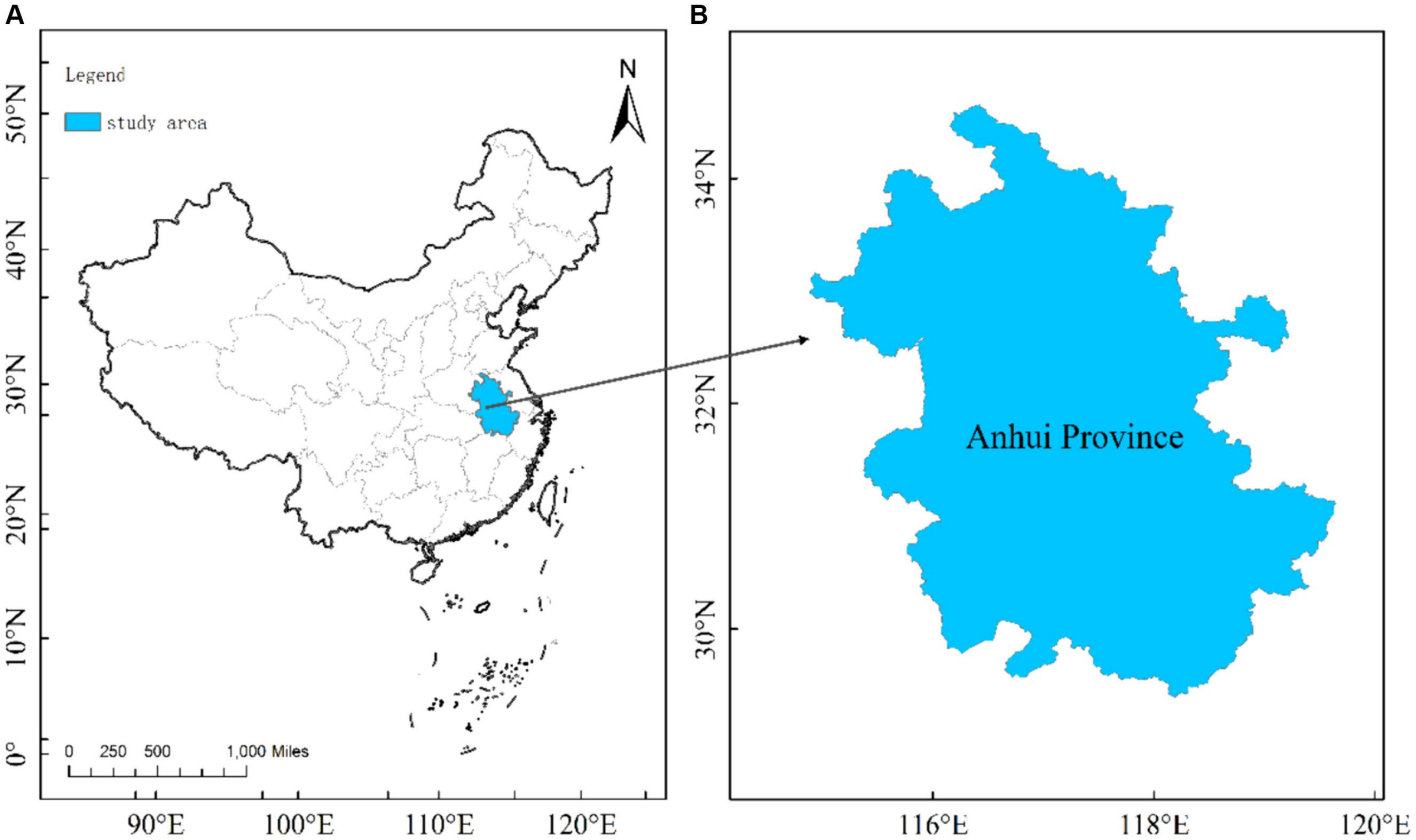
Geographical location **(A)** and map display **(B)** of Anhui Province, China.

Anhui Province, located in the eastern part of China (Figure 1A), is an epidemic area of HFRS Figure 1B is the map of Anhui Province, which spans 450 kilometers from east to west and 570 kilometers from north to south, covering a jurisdictional area of 140,100 square kilometers, of which 139,400 square kilometers is land. Anhui features a diverse range of landforms, including plains, plateaus, hills, and mountains, and is home to a population of 61.27 million residents. The annual average temperature in Anhui ranges from 14 to 17°C, while the annual average precipitation varies from 773 to 1,670 millimeters, with abundant rainfall during the summer months, accounting for 40–60% of the total annual precipitation.

### 2.2 Data collection

#### 2.2.1 The HFRS dataset

The monthly HFRS data collected in this study, covering the period from 2005 to 2019 in Anhui Province, are obtained from the Chinese Center for Disease Control and Prevention’s Public Health Science Data Center (CCDC). All disease surveillance data are anonymized, and all included patients meet the diagnostic criteria and management principles for HFRS established by the Ministry of Health of the People’s Republic of China.

#### 2.2.2 Satellite and reanalysis datasets

Several auxiliary datasets are utilized to investigate the impact of external environment and climate on the development of HFRS (Table 1). The satellite datasets include the 16-day composite NDVI product (MOD13Q1) and the daily AOD product (MCD19A2), both derived from the Moderate Resolution Imaging Spectroradiometer (MODIS), which are recognized for their high accuracy globally, including in China (17–19). The MCD19A2 dataset provides AOD measurements at two wavelengths, namely blue wavelength at 470 nm and green wavelength at 550 nm. For this study, we select the highest quality MAIAC AOD data at the 550 nm wavelength to minimize the influence of outliers. The MOD13Q1 employs the maximum value composites (MVC) method to generate the monthly NDVI data, aiming to improve the accuracy of NDVI data (20). All MODIS products used in this study are accessible through the NASA website.^1^

**Table 1:** Overview of the climatic and environmental datasets used in this study.

	Variables	Database	Spatial resolution	Annotations
Satellite products	AOD	MCD19A2	1 km	Aerosol Optical Depth
	NDVI	MOD13Q1	250 m	Normalized Difference Vegetation Index
Reanalysis products	P	ERA5-Total precipitation	0.25°	Precipitation
	RH	ERA5-Relative humidity	0.25°	Relative Humidity
	SR	ERA5-Surface Solar radiation downwards	0.1°	Solar radiation
	T (Tmin and Tmax)	ERA5-2 m temperature	0.25	Mean Temperature (Minimum and Maximum Temperature)

The reanalysis datasets are sourced from ERA5, the fifth generation ECMWF atmospheric reanalysis of the global climate, which integrates model data with observational data from around the world using physical laws and offers a comprehensive representation of the global climate from 1950 to the present (21, 22). These datasets offer advantages such as high spatio-temporal resolution, a wide range of variables, and rapid update speeds, making them a valuable resource for diverse research and applications (23, 24). This study utilizes four variables from ERA5, including total precipitation, relative humidity, surface solar radiation downwards, and 2 m air temperature (25). All satellite and reanalysis datasets covering the period from 2005 to 2019 are used to synthesize monthly average data for Anhui Province.

### 2.3 Statistical analysis

#### 2.3.1 Wavelet analysis

Wavelet analysis is a versatile tool for time-series analysis, providing insights into both the temporal and frequency characteristics of signals (26–28). This technique is particularly effective in identifying non-stationary features within a signal and has been widely applied across various fields. In this study, continuous wavelet transform (CWT) is employed to detect periodic fluctuations in the monthly cases of HFRS in Anhui Province from 2005 to 2019. The CWT operates by performing an inner product operation on the original time-domain signal x(t) with a chosen mother wavelet *ψ*(t), resulting in the decomposition of wavelet transform coefficients *λ*(s, t). This process constructs a time-frequency signal with good localization in both time and frequency domains, defined as Equation 1 (29):


(1) $$\lambda \left(s,t\right)=\int x\left(t\right)\psi_{s,\tau }^{*}\left(t\right)dt$$

Where ψ^*^_s,τ_(t) represents the conjugate operations of the wavelet basis functions ψ_s,τ_(t).

#### 2.3.2 Spearman correlation analysis

Preliminary analysis indicated that the data do not follow a normal distribution and May lack a linear relationship between variables. As a result, a nonparametric method is chosen for the analysis. Specifically, we employ Spearman’s rank correlation analysis, where the relationship between variables x and y is determined by the Equation 2 (30):


(2) $$p=\frac{\sum_{i}x_{i}-\bar{x}y_{i}-\bar{y}}{\sqrt{\sum_{i}x_{i}-{\bar{x}}^{2}\sum_{i}y_{i}-{\bar{y}}^{2}}}$$

#### 2.3.3 Poisson regression analysis

In general, the occurrence of HFRS is a low-probability event, and as a type of time series data, it approximates a Poisson distribution. In this study, a time-series-based Poisson regression model is employed to investigate the association between monthly HFRS cases and various climatic (e.g., P, T, Tmin, Tmax, RH, and SR) and environmental (e.g., AOD and NDVI) factors in Anhui Province from 2005 to 2018. The model’s effectiveness is evaluated using the monthly incidence data from an independent year, specifically 2019 (31, 32).

The generalized linear model is represented by the Equation 3:


(3) $$\eta =g\left[\mu \left(Y\right)\right]=\beta_{0}+\beta_{1}X_{1}+\beta_{2}X_{2}+\ldots +\beta_{j}X_{j}$$

η is known as the connectivity function and $g\left[\right]$ represents a specific function of μ . After expressing $g\left[\right]$ in its logarithmic form, the formula is then transformed into its exponential form, resulting in Equation 4:


(4) $$\ln\left(Y\right)=\beta_{0}+\beta_{1}X_{1}+\beta_{2}X_{2}+\ldots +\beta_{j}X_{j}$$

When $X_{j}$ changes by one unit, the multiple of the predicted count, known as the relative risk (RR), is given by $\exp\left(\beta_{j}\right)$ . This relationship is expressed in Equation 5:


(5) $$RR\left(Y\right)=\exp\left(\beta_{1}X_{1}+\beta_{2}X_{2}+\ldots +\beta_{j}X_{j}\right)$$

To account for the lagged and seasonal effects of climatic and environmental factors on HFRS incidence, lagged variables (1–6 months) are incorporated into the original Poisson regression model, resulting in the development of a time-series-based regression model which is described in Equation 6:


(6) $$\ln\left(Y_{t}\right)=\beta_{0}+\beta_{1}X_{1\left(t-n\right)}+\ldots +\beta_{j}X_{i\left(t-n\right)}$$

Where $Y_{t}$ is the number of monthly HFRS cases, $\beta_{j}$ is the partial regression coefficient, t is the month, n is the lag period, and $X_{i\left(t-n\right)}$ represents the lag period-adjusted climatic and environmental factors. This study uses the variance inflation factor (VIF) to evaluate the multicollinearity among explanatory variables, selects suitable candidates for Poisson analysis, and then applies the Akaike information criteria (AIC) to test the model’s goodness of fit (12, 33). The Pearson correlation coefficient (R) and the root mean squares error (RMSE) are selected as metrics to evaluate the correlation and error between fitted and actual values, respectively (34–36). Furthermore, an F-test is employed to statistically determine whether a significant difference exists between aforementioned two datasets. All these analyses are conducted using MATLAB software.

## 3 Results

### 3.1 Characteristics of monthly HFRS incidence from 2005 to 2019

**Figure 2:**
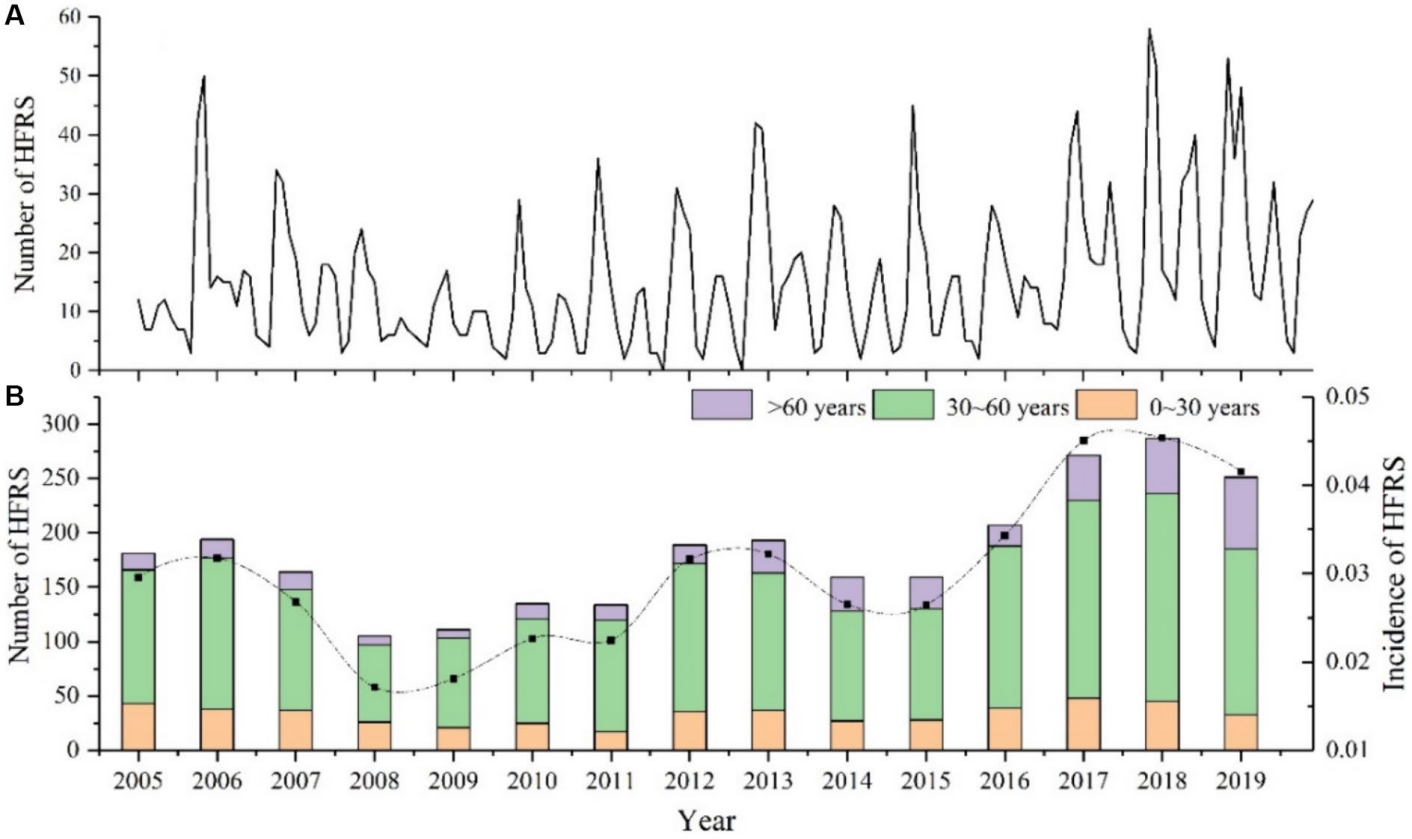
Epidemiological Features of HFRS in Anhui Province from 2005 to 2019. **(A)** Monthly number of HFRS cases. **(B)** Annual incidence rate and age distribution.

From 2005 to 2019, a total of 2,744 HFRS cases are reported in Anhui Province, Figure 2A displays the monthly number of HFRS cases, it can be observed that the incidence of HFRS follows a clear seasonal pattern, with peaks generally occurring in autumn (September to November) and winter (December to February of the following year), accounting for 31.79 and 30.94% of cases, respectively. This is followed by an incidence rate of 20.3% in spring (March to May), while the incidence rate decreases significantly in summer (June to August), reaching only 16.98%. In terms of annual characteristics (Figure 2B), there is a significant increase from 2005 to 2006, followed by a decline from 2007 to 2008, with the annual incidence rate decreasing to 0.017 per 100,000. From 2015 to 2018, a small peak in the number of cases is observed, reaching 0.045 per 100,000 in 2018, the highest incidence rate during the study period. Notably, the age group of 30 to 60 years old consistently shows a higher incidence rate than other age groups, accounting for more than 50% of all cases each year (ranging from 55.73 to 74.63%). Among them, the proportion of cases in this age group reach the lowest at 55.73% in 2019, while it peak at74.63% in 2012. Since 2013, there is an increasing trend in the incidence rate among people over 60 years old, while the incidence rate among those under 30 years old remain relatively stable in most years.

**Figure 3:**
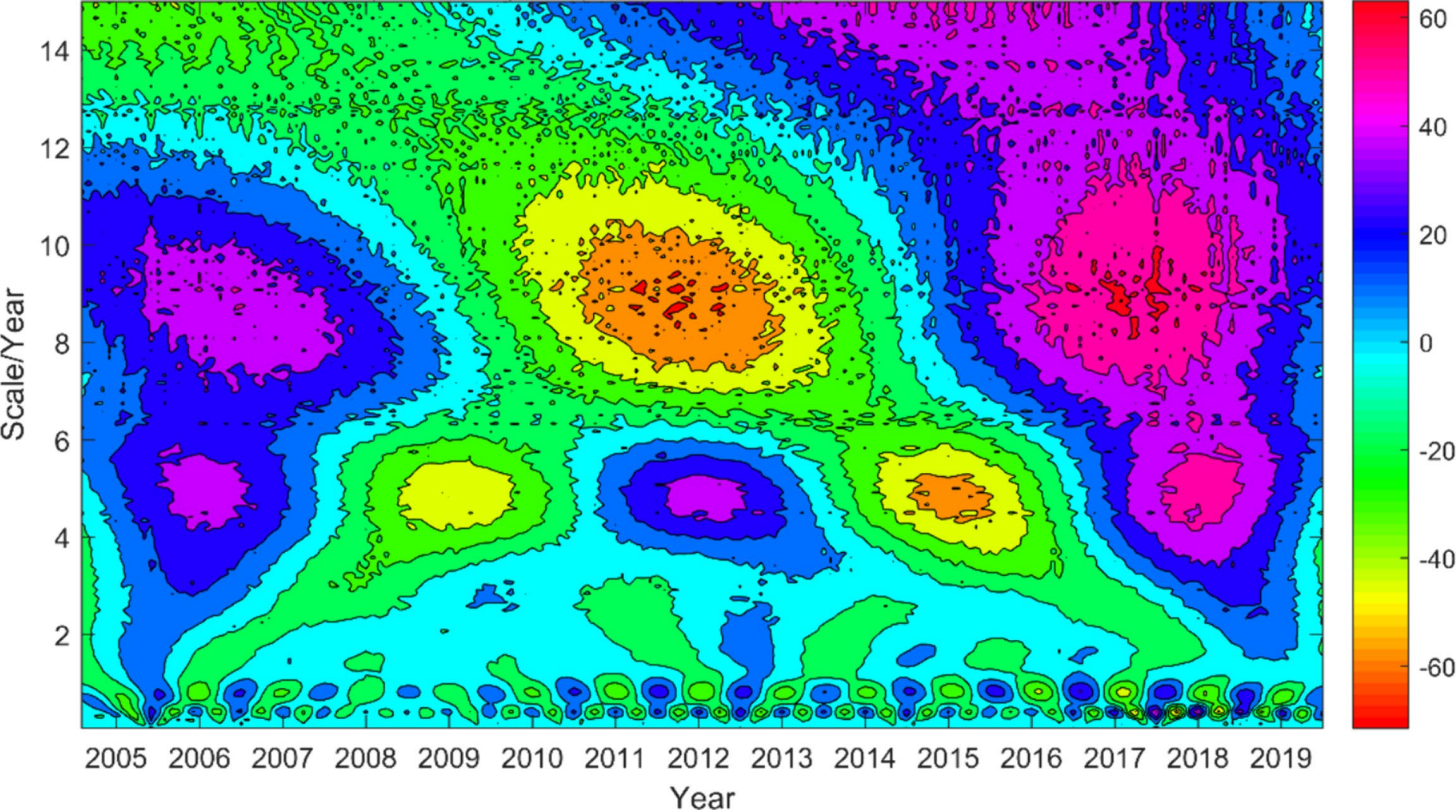
Wavelet coefficient real part contour map of the incidence number series of HFRS from 2005 to 2019.

To gain a better understanding of the periodicity of HFRS cases in Anhui Province, we conducted a Morlet wavelet analysis on the incidence of HFRS from 2005 to 2019. By plotting the contour map of the real part of the wavelet coefficients (Figure 3), it can be observed that the number of HFRS cases exhibits periodic variations at three time scales: 0.5–1 year, 4–6 years, and 7–11 years. While the oscillation at the 0.5–1 year time scale is less pronounced compared to the other two, it persists throughout the entire study period and shows a higher frequency and complexity in its oscillation cycles. Additionally, the oscillation periods at the 4–6 year and 7–11 year time scales are more evident, with each exhibiting 2.5 and 1.5 cycles of “high-low” disease incidence, respectively.

**Figure 4:**
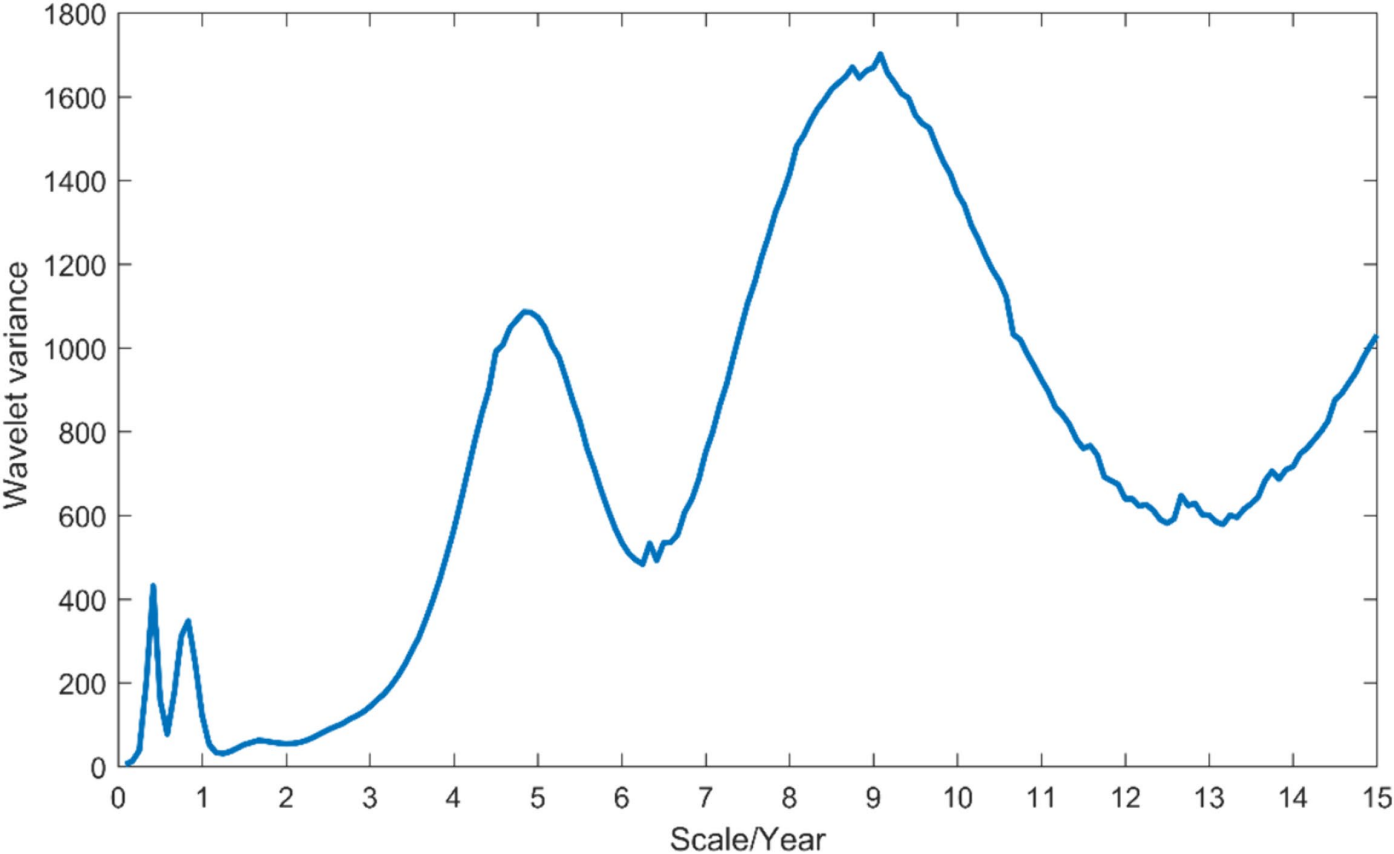
Wavelet variance plot of monthly HFRS incidence number series in Anhui Province.

By applying the formula for wavelet coefficients and wavelet variance, the wavelet variances at different time scales are calculated to reveal the distribution of energy in the time series (Figure 4). From 2005 to 2019, three distinct peaks are observed, corresponding to time scales of approximately 0.5 years, 5 years, and 9 years. The peak with the most obvious intensity is observed in the 9-year cycle, indicating the strongest energy and maximum cyclic oscillations. The 5-year cycle shows a secondary peak, while the 0.5-year cycle, representing the shorter cycle, has the weakest peak. These three cycles reflect the changing characteristics of HFRS incidence in Anhui Province over the study period.

### 3.2 Spearman correlation analysis

**Figure 5:**
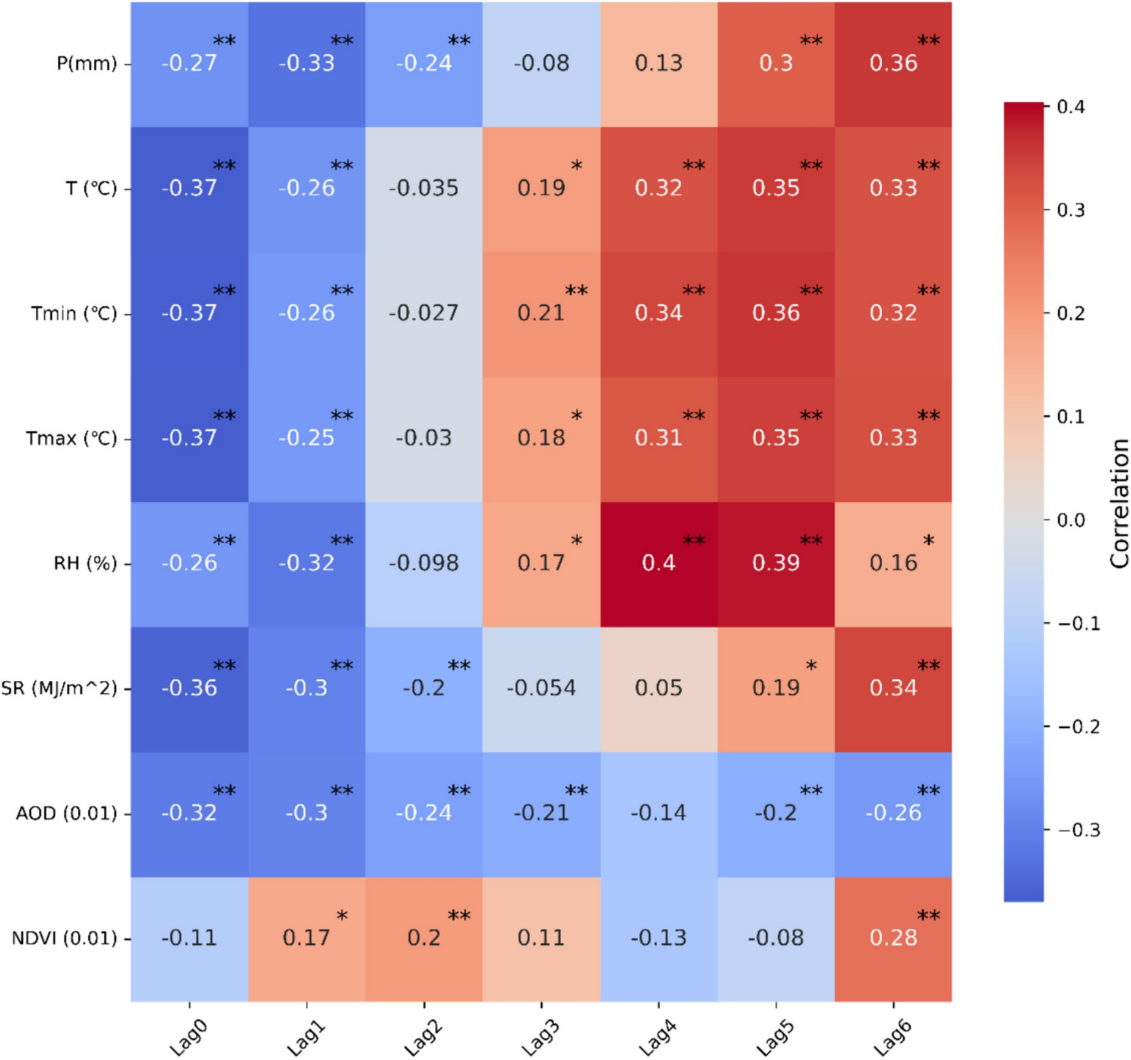
Spearman’s correlation heat map between monthly incidence of HFRS and each climatic and environmental factor in Anhui Province, China. ^**^ Indicates a significant correlation at the 0.01 level of significance, ^*^ indicates a significant correlation at the 0.05 level of significance.

A Spearman correlation analysis is first conducted between the incidence of HFRS and each climate and environmental factor in Anhui Province from 2005 to 2019 (Figure 5). The results indicate a close correlation between the number of HFRS cases and changes in climate and environmental conditions, with varying degrees of association. Both P and SR show a consistent pattern, exerting negative impact on HFRS prevalence in the three preceding months and a positive impact in the 4-6-month lag period, reaching peak positive correlation coefficients at a 6-month lag. Notably, the correlation coefficient for AOD remains consistently negative, with the maximum correlation coefficient observed in the current month or within 1-month lag. Interestingly, all temperature-related factors, including monthly average temperature, maximum temperature, and minimum temperature, shift from a negative to a positive correlation as the lag time progresses, and the correlation coefficients gradually increase. The greatest impact on the incidence of HFRS is observed at a 5-month lag, which is consistent with the findings of Li et al. (12). This is likely because temperature significantly influence rodent population density and hantaviruses infection rates, both of which are affected by seasonal changes (37, 38). Regarding NDVI, it exhibits relatively low correlation coefficients and lacks statistical significance across multiple time periods (*p* > 0.05), possibly because NDVI May not directly reflect the key factors related to the transmission and incidence of HFRS in Anhui. These findings underscore the importance of considering appropriate lag times for climatic and environmental variables in order to enhance the accuracy of predictive models and improve strategies for preventing of HFRS outbreaks.

### 3.3 Result of Poisson regression analysis

#### 3.3.1 Single-factor correlation analysis

**Figure 6:**
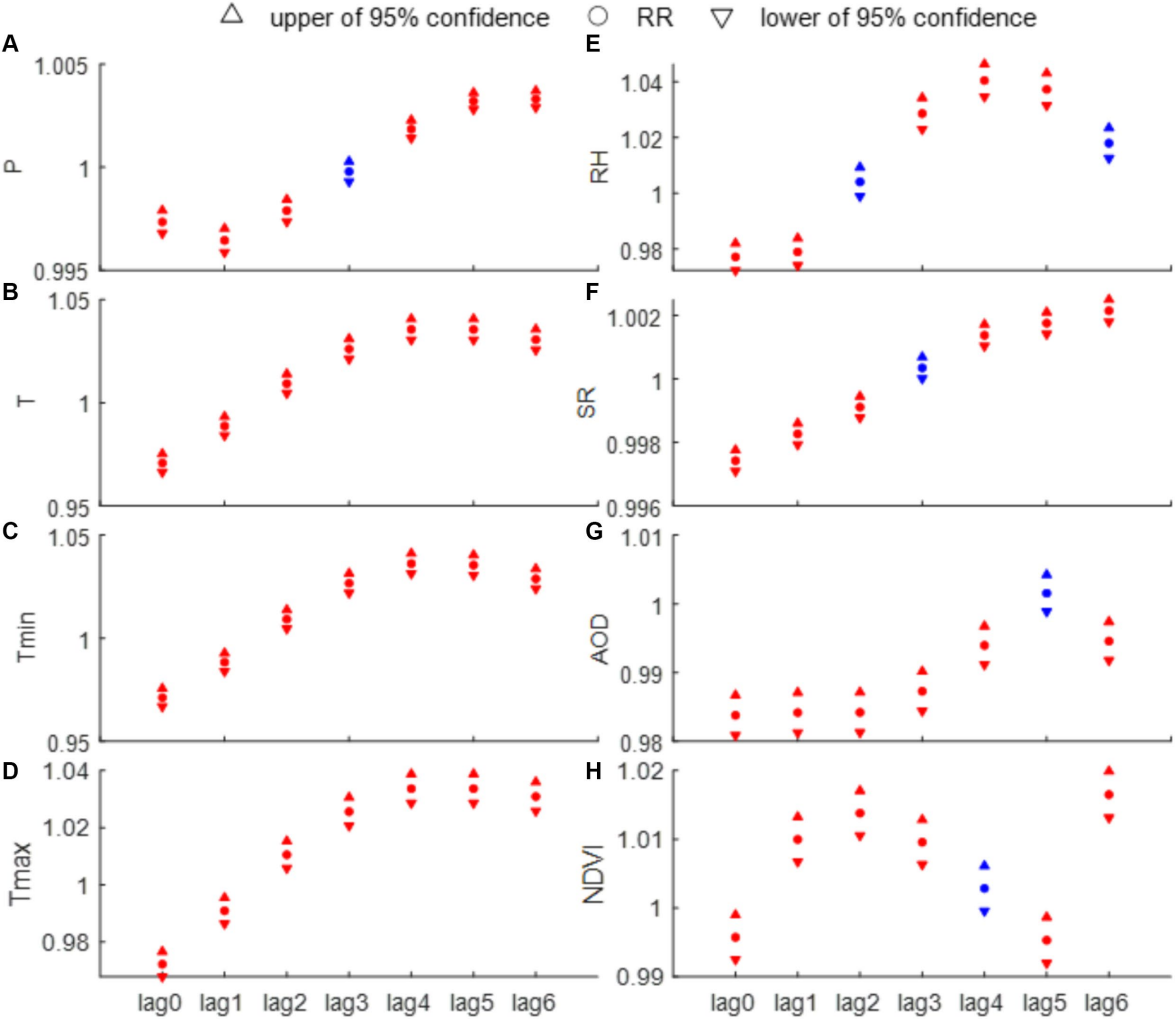
Results of univariate analysis of the number of HFRS incidence and each influencing factor [**(A)** P, **(B)** T, **(C)** Tmin, **(D)** Tmax, **(E)** RH, **(F)** SR, **(G)** AOD, and **(H)** NDVI]. The red solids represent a statistically significant difference (*p* < 0.01), while the blue solids indicate the opposite.

To further understand the epidemiological characteristics of HFRS, this section investigates the effect of individual factor on the monthly HFRS cases through Poisson regression analysis. The results indicate that all eight factors (Figures 6A–H) and their associated lag variables are statistically associated with HFRS cases in most instances (*p* < 0.1). Interestingly, temperature-related indicators (i.e., T, Tmax, and Tmin) have statistical significance at lag times of 0–6 months. At a 4-month lag, each 1°C increase in Tmin is associated with a 3.55% increase in HFRS cases (95% CI, 3.08–4.03%), and each 1% increase in RH, there is a 3.97% increase in HFRS cases (95% CI, 3.41–4.53%). At a lag time of 6 months, for every 1 mm increase in precipitation corresponds to a 3.3% rise in HFRS cases (95% CI, 0.29–0.37%). Additionally, a 0.01 increase in NDVI is linked to a 1.64% increase in HFRS cases (95% CI, 1.3–1.97%). It can be noted that T and Tmax have the same RR at lag times of 4 and 5 months, both reaching the highest risk for HFRS incidence. Conversely, SR and AOD present the lowest relative risks in the same month (Figure 6).

#### 3.3.2 Attribution analysis based on Poisson regression

**Figure 7:**
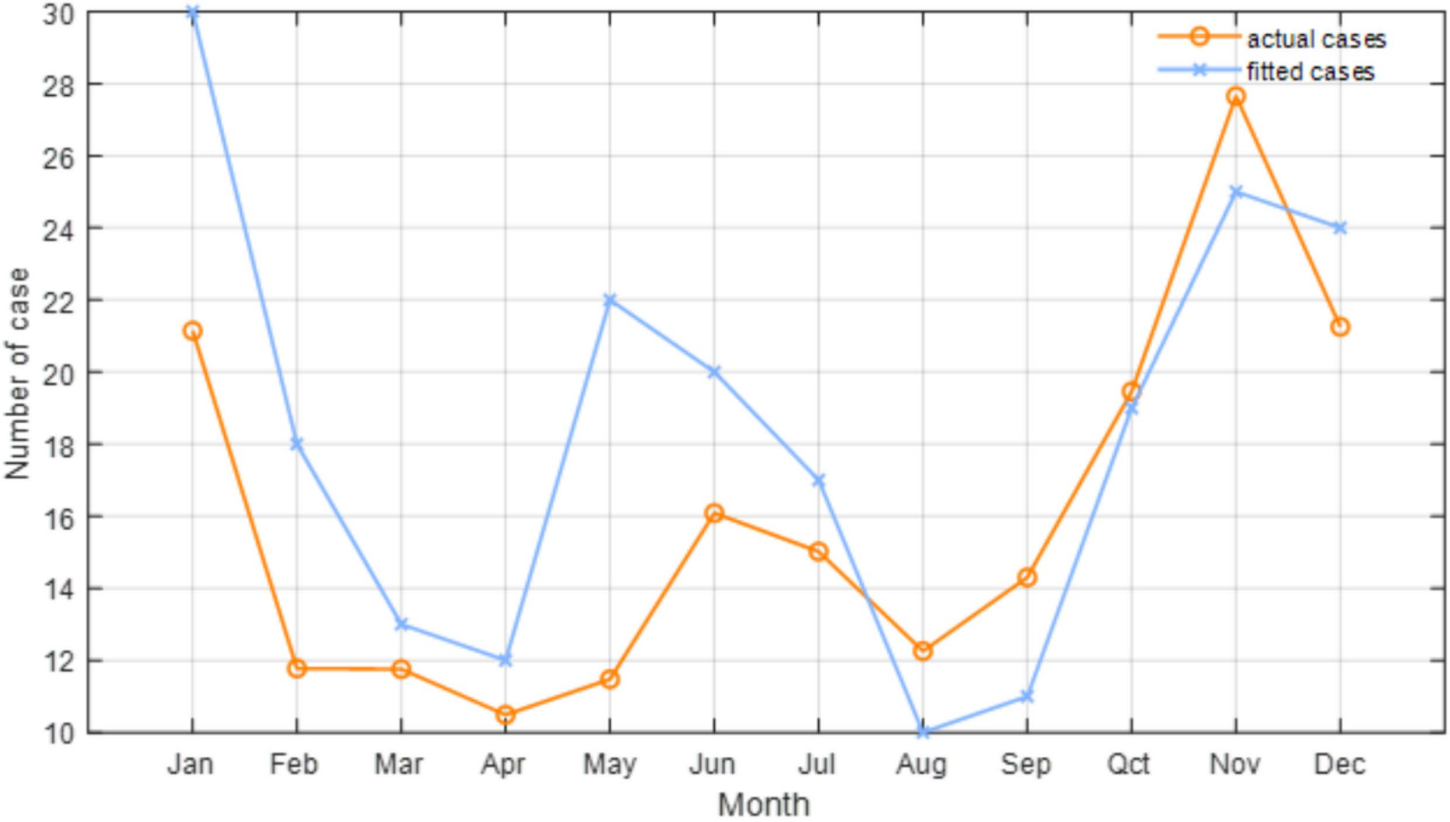
Relationship between the number of HFRS cases and the Poisson regression fitted in Anhui Province in 2019.

After evaluating potential model predictors using the VIF test, we identified significant multicollinearity among the temperature-related variables (T, Tmin and Tmax), which impacts the model’s explanatory capacity. Considering this, along with findings from previous studies (9, 10, 39), we choose to retain the primary temperature variable T, while excluding Tmin and Tmax. The VIF values of the retained factors after this exclusion are all less than 10. Consequently, based on the AIC, we finalize the selection of climatic (e.g., P, T, RH, and SR) and environmental (e.g., AOD and NDVI) factors, along with their corresponding under specific lag periods (Table 2). Specifically, the included factors are lagged P at 5 months, lagged T at 4 months, lagged RH at 5 months, lagged SR at 4 months, lagged AOD at 5 months, and lagged NDVI at 2 months. Figure 7 shows the relationship between the cases estimated by the Poisson regression model and the actual cases for each month in 2019. The evaluation indicates that the final model we constructed fits the actual cases well, although there are some discrepancies between the fitted and actual cases. For example, the fitted cases are higher than the actual cases from January to July, while the fitted cases from August to November are lower than the actual cases. Despite this, the R and RMSE of the predictive model, compared to the actual data, reach 0.887 and 11, respectively. Furthermore, the F-test shows that there is no significant difference between the actual and fitted cases (*p* = 0.67), suggesting the robustness of the constructed model. Moreover, our final Poisson regression results suggest that a 1 mm increase in monthly P May be associated with an 0.14% increase in HFRS cases (95% CI, 0.07–0.21%). Importantly, temperature has the most significant impact on HFRS cases. For every 1°C increase in monthly T, HFRS cases increase by 4.22% (95% CI, 3.22–5.23%) per month. In addition, RH and NDVI are found to exert positive influences on the cases of HFRS, whereas SR and AOD have opposite effects.

**Table 2:** Parameters estimated by Poisson regression analysis of climatic and environmental factors in the cases of HFRS.

Variable	RR	95%CI	*p*-value
P, 5-month lag	1.0014	(1.0007, 1.0021)	0.0001
T, 4-month lag	1.0422	(1.0322, 1.0523)	0.0000
RH, 5-month lag	1.0080	(1.0000, 1.0161)	0.0491
SR, 4-month lag	0.9981	(0.9975, 0.9987)	0.0000
AOD, 5-month lag	0.9967	(0.9940, 0.9993)	0.0123
NDVI, 2-month lag	1.0120	(1.0086, 1.0154)	0.0000

## 4 Discussion

In this study, a substantial correlation is identified between the number of HFRS cases and various meteorological and environmental factors. Consistent with previous research, a higher incidence rate of HFRS is observed in Anhui Province during autumn and winter, followed by spring (40–43). Given the known peak seasons for hantavirus outbreaks in specific rodent types, such as apodemus in autumn and winter, house mice in spring, and mixed types experiencing outbreaks across multiple seasons (40), it is inferred that Anhui May represent a mixed epidemic area. The cold winter conditions encourage rodents to gather in residential areas, leading to increased population density and closer contact with humans, creating a favorable environment for hantavirus transmission (16). However, considering the lag effect of diseases, focusing solely on rodent control during the high infestation periods of autumn and winter is insufficient (44). Overall, the seasonal variations in HFRS incidence highlight the vital role of climatic and environmental factors in disease transmission.

In comparison to other age groups, the 30–60 age group exhibits a significantly higher incidence rate of HFRS. This pattern May be attributed to the increased engagement in outdoor labor among individuals in this age bracket, which elevates their risk of viral exposure and HFRS infection (40). This emphasizes middle-aged individuals as a high-risk and susceptible population for HFRS (16). Between 2017 and 2019, there has been a slight increase in the incidence rate of HFRS among individuals aged 60 and above, while a slight decrease has been observed among younger people. This shift May be associated with more young and middle-aged individuals participating in outdoor labor, leading to greater exposure to wild rodents and their excreta. Consequently, the expanded vaccination program in key areas for young and middle-aged individuals has likely contributed to elevated immune levels against HFRS in this population. Furthermore, our analysis reveals three distinct cycles within the HFRS cases data, with the most pronounced peak in wavelet variance corresponding to the 9-year cycle, indicating significant periodic fluctuation in HFRS cases (45). The application of wavelet analysis in this study not only uncovers the underlying periodicity in HFRS incidence but also paves the way for a more nuanced investigation into the interplay between climatic variables and disease transmission dynamics (46).

It is noteworthy that climate change can significantly impact the transmission dynamics of HFRS (2). The monthly HFRS cases exhibit a shift from negative to positive correlation with P over a lag range of 0–6 months, showing the most significant impact at a 6-month lag. Increased precipitation can directly or indirectly influence vegetation growth, providing a food source for rodent hosts and subsequently leads to an increase in rodent population density (37, 47). However, during periods of continuous heavy rainfall or flooding, excessive precipitation or flooding May have destructive effects on rodent habitats, reducing rodent mobility and, consequently, lowering the risk of human contact with rodents (43). Temperature and humidity also play pivotal roles in influencing rodent activity, hantavirus movement, and infectivity. Warm and humid climates can prolong the survival time of rodents, increase the number of infectious rodents throughout their lifecycle, and enhance the spread and persistence of hantaviruses (2).

Given that solar radiation and sunshine duration are strongly correlated (42), SR is used in this study as an indirect measure of sunshine duration. The number of HFRS cases shows a negative correlation with SR in the current month and at lags of 1–2 months, while a positive correlation is observed at lags of 5–6 months. Increased SR or prolonged sunshine duration, coupled with increased outdoor human activity, May exacerbate the spread of HFRS. The AOD value used in this study can, to some extent, represent air quality and the lag effect of AOD on HFRS risk aligns with previous research findings (47). Additionally, NDVI is employed to reflect the vegetation growth status and coverage level, and the higher the value, the better the vegetation growth. Multiple studies have shown that there is a certain correlation between NDVI and the spread of HFRS (4, 48, 49). NDVI can even serve as an indicator food availability for rodent hosts (37). However, in our study, NDVI does not show a statistically significant impact on HFRS across multiple time periods, which May be related to the fact that NDVI values are not obtained from the specific locations of patients. Despite this, vegetation factors should not be overlooked when formulating prevention and control measures (4).

In fact, the developed model for predicting HFRS incidence provides a valuable tool for relevant authorities to plan and issue timely warnings, and implement public health interventions. However, it is essential to acknowledge certain limitations in this study. Firstly, the research scope is limited to the entire Anhui Province due to the lack of detailed HFRS data, and the nuanced differences in meteorological and environmental factors across cities and counties have not been fully considered, which May introduce uncertainties in the research results. Secondly, the reliance on passive surveillance data from the CCDC for case numbers May result in the oversight of unreported clinically asymptomatic cases, potentially underestimating the true incidence rate. Lastly, the predictive model developed in this study primarily focuses on short-term forecasts, limiting its ability to capture long-term trends in HFRS incidence. Furthermore, other factors, such as rodent density, socio-economic variables, and disease prevention measures play crucial roles in HFRS transmission and should be incorporated into future research to provide a more comprehensive analysis of the relationship between HFRS and its influencing factors.

## 5 Conclusion

The research findings underscore the pivotal roles of climatic and environmental factors in influencing the transmission of HFRS in Anhui Province, China. These factors what we found exert an impact on both the viral and rodent transmission of HFRS. It is crucial, especially in climate change-prone regions, to promptly establish early warning systems and implement effective public health measures to mitigate potential outbreaks. Early warning systems based on meteorological forecasts can enhance the prediction of HFRS incidence, offering valuable insights for timely interventions and the formulation of prevention strategies.

## Data Availability

The raw data supporting the conclusions of this article will be made available by the authors, without undue reservation.

## Footnotes

^1^ https://search.earthdata.nasa.gov/
